# Use of Gene Expression Profiles of Peripheral Blood Lymphocytes to Distinguish *BRCA1* Mutation Carriers in High Risk Breast Cancer Families

**DOI:** 10.4137/cin.s931

**Published:** 2009-03-02

**Authors:** Marie-Laure Vuillaume, Nancy Uhrhammer, Véronique Vidal, Valérie Sylvain Vidal, Valérie Chabaud, Beline Jesson, Fabrice Kwiatkowski, Yves-Jean Bignon

**Affiliations:** Département d’Oncogénétique, Centre Jean Perrin, Clermont-Ferrand, France (MLV, NU, YJB, FK); Diagnogène S.A., biopôle Clermont-Limagne, France (VV, VS, VC, BJ)

**Keywords:** microarray, gene expression profile, peripheral blood mononuclear cells, hereditary breast cancer, BRCA1, molecular genetic diagnostics

## Abstract

Mutations in two major genes, *BRCA1* and *BRCA2*, account for up to 30% of families with hereditary breast cancer. Unfortunately, in most families there is little to indicate which gene should be targeted first for mutation screening, which is labor intensive, time consuming and often prohibitively expensive. As *BRCA1* is a tumor suppressor gene involved in various cellular processes, heterozygous mutations could deregulate dependent pathways, such as DNA damage response, and disturb transcriptional activity of genes involved in the downstream signaling cascade. We investigated gene expression profiling in peripheral blood lymphocytes to evaluate this strategy for distinguishing *BRCA1* mutation carriers from non-carriers. RNA from whole blood samples of 15 *BRCA1* mutation carriers and 15 non-carriers from *BRCA1* or *BRCA2* families were hybridized to Agilent Technologies Whole Human Genome OligoMicroarrays (4 × 44 K multiplex format) containing 41,000 unique human genes and transcripts. Gene expression data were analyzed with Welch’s t-tests and submitted to hierarchical clustering (GeneSpring GX software, Agilent Technologies). Statistical analysis revealed a slight tendency for 133 genes to be differentially expressed between *BRCA1* mutation carriers and non-carriers. However, hierarchical clustering of these genes did not accurately discriminate *BRCA1* mutation carriers from non-carriers. Expression variation for these genes according to *BRCA1* mutation status was weak. In summary, microarray profiling of untreated whole blood does not appear to be informative in identifying breast cancer risk due to *BRCA1* mutation.

## Introduction

Breast cancer is the most common cancer in women in the western world, of which approximately five to ten percent of cases are of hereditary origin. Two major susceptibility genes, *BRCA1* and *BRCA2*, were identified through positional cloning in 1994^1^ and 1996^2^ respectively. Mutations in these genes account for up to 30% of families with hereditary breast cancer. These genes are risk factors with by far the highest predictive value, and they may be targeted for analysis according to the familial phenotype. *BRCA1* mutations are associated with female breast and ovarian cancer, while *BRCA2* mutations are rather associated with female and male breast cancer and to a lesser extent with ovarian cancer. Despite these differences in familial phenotype, the majority of families present only early onset breast cancer and there is little to indicate which gene should be targeted first for more efficient mutation screening or if in fact one of the *BRCA1* or *BRCA2* gene is at cause.

*BRCA1* is a tumor suppressor gene involved in various cellular processes, notably DNA damage response, cell cycle control, chromatin remodeling, ubiquitination and transcriptional regulation.^3^,^4^ The involvement of *BRCA1* in these processes is highlighted by its interaction with a variety of proteins, including DNA damage repair proteins (RAD50, RAD51, *BRCA2*, MLH1, FANCA), transcriptional activators and repressors (RNA polymerase II, RNA helicase A, histone deacetylase 1, CtBP1, ERalpha, AR, STAT1) and cell cycle checkpoint proteins (p53, cyclins and cyclin dependent kinases).^5^ Microarray studies have shown that *BRCA1* transcriptionally regulates genes involved in breast tumorigenesis, most notably those coding for p21^WAF1/CIP1^, GADD45, 14-3-3σ, c-Myc and cyclin D1.^6^ Hemizygosity for *BRCA1* could thus have an effect on expression levels of these genes.

Microarray studies have also shown that constitutional mutations in *BRCA1* and *BRCA2* influence the gene expression profile of malignant tissues.^7^–^13^ In primary tumors from breast epithelium, Hedenfalk et al. showed that there are different gene expression profiles in *BRCA1* positive tumors, *BRCA2* positive tumors and sporadic tumors.^8^ Comparison of gene expression patterns in ovarian cancers showed that *BRCA1* and *BRCA2* associated tumors differ significantly in their gene expression profiles.^1^, ^2^

With regard to healthy tissues, studies of fibroblasts cultured from breast^14^ and skin biopsies^15^ showed that irradiated cells from heterozygous *BRCA1* mutation carriers display gene expression profiles different from those of non-carriers^14^ and those of *BRCA2* mutation carriers.^15^ These results demonstrate the involvement of BRCA1 and BRCA2 in DNA damage response and the potential existence of a distinct functional heterozygous phenotype for *BRCA1* carriers. This hypothesis was assessed through studies of irradiated human lymphocytes from heterozygous *BRCA1* and *BRCA2* mutation carriers.^16^–^18^ These studies analyzed the cellular phenotype of irradiated lymphocytes and showed a deficit in DNA damage response resulting in micronuclei formation in irradiated G0 cells^17^,^18^ and in an increased level of chromosomal aberrations after irradiation.^16^

These different studies show that gene expression profiles associated with *BRCA1* or *BRCA2* mutation status can be found in malignant tissues and in irradiated healthy tissue. However, these two approaches cannot be easily applied to diagnostic screening: the first case requires a tumor sample and the second case requires irradiation (or treatment with other DNA damaging agents) of fresh lymphocytes or cell lines. We therefore proposed to examine gene expression profiles of *BRCA1* mutation carriers and *BRCA1* or *BRCA2* non-carriers in an accessible tissue such as peripheral blood mononuclear cells (PBMCs). Our aim was to assess if a *BRCA1*-carrier profile could be identified in untreated samples. If so, this profile could allow the development of a test for flagging likely *BRCA1* mutation carriers. The interest of working with untreated samples is the broader range of samples accessible for testing, notably those drawn at distant locations and sent to the laboratory by mail. The routine treatment of such samples with DNA damaging agents in a timely and homogeneous manner would not be practical.

The use of untreated PBMCs is relevant in light of the established links between DNA damage response, immunity and cancer.^19^ Other studies have successfully used PBMCs to demonstrate that breast cancer affects gene expression patterns in peripheral blood cells during early stages of disease development.^20^ Inter-individual variation observed in peripheral blood^21^–^23^ was shown to be minimal in comparison to that observed associated with various diseases and disorders^21^,^23^ such as cancer or infectious disease.

In the present study, we compared gene expression profiles in peripheral blood cells of *BRCA1* mutation carriers who belong to high-risk breast cancer families with gene expression profiles of *BRCA1* or *BRCA2* mutation non-carriers in order to evaluate the possibility of setting up a microarray-based preliminary screening tool.

## Materials and Methods

### Case selection criteria

All samples were taken from members of high-risk breast cancer families ascertained through the Oncogenetic consultation at the Centre Jean Perrin. Individuals were asked to provide a blood sample and to sign an informed consent form approved by the CCPPRB regional ethics committee (Auvergne). Fifteen samples from patients with germline mutations of *BRCA1* and fifteen samples from family members without the familial mutation were selected for analysis. Mutation screening was performed by direct sequencing.

### RNA isolation

Peripheral Blood Mononuclear Cells (PBMCs) were isolated on a density gradient. Briefly, 3 ml of Pancoll (PAN Biotech GmbH, Aidenbach, Germany) was added to a LeucoSep tube (Dutscher, Brumath, France) and centrifuged to position the porous LeucoSep membrane on the Pancoll surface. Approximately 6 ml of heparinized blood was poured onto the membrane, and the tubes were centrifuged at 1000 g for 10 min at room temperature. After centrifugation, the interface containing PBMCs was collected and washed twice with PBS (Invitrogen, Carlsbad, CA). Total RNA was extracted with TRIzol reagent (Invitrogen, Carlsbad, CA) according to the manufacturer’s instructions. RNA quantity and quality were determined using the RNA 6000 Nano Assay kit on an Agilent 2100 BioAnalyzer (Agilent Technologies, Palo Alto, CA), as recommended. A commercial pool of total RNA (ref. 636580 BD Biosciences Clontech, Heidelberg, Germany) extracted from normal human peripheral leukocytes of 13 healthy male/female Caucasians was used as a reference RNA cohybridized with the test sample (carriers or controls) in each microarray.

### cRNA amplification and labeling

Total RNA was amplified and labeled with Cyanine 5 for test samples (carriers and controls) and with Cyanine 3 for the reference using Agilent’s Low RNA Input Linear Amplification Kit (Agilent Technologies, Palo Alto, CA) following the detailed protocol described in the kit manual (Manual Part Number G4140-90050 version 5.0.01). Briefly, 1 μg of total RNA was reversed transcribed to double-strand cDNA using a poly dT-T7 promoter primer. Primer, template RNA and quality-control transcripts of known concentration and quality were first denatured at 65 °C for 10 min and incubated for 2 hours at 40 °C with 5X first strand Buffer, 0.1 M DTT, 10 mM dNTP, MMLV RT, and RNase-out. The MMLV RT enzyme was inactivated at 65 °C for 15 min. cDNA products were then used as templates for *in vitro* transcription to generate fluorescent cRNA. cDNA products were mixed with a transcription master mix in the presence of T7 RNA polymerase and CY5 labeled or CY3 labeled-CTP and incubated at 40 °C for 2 hours. Labeled cRNAs were purified using QIAGEN’s RNeasy mini spin columns and eluted in 30 μl of nuclease-free water. After amplification and labeling, cRNA quantity and cyanine incorporation were determined using a nanodrop ND.1000 UV-VIS-Spectrophotometer version 3.2.1(Agilent Technologies, Palo Alto, CA).

### Sample hybridization

For each hybridization, 825 ng of Cyanine 3 labeled cRNA (reference) and 825 ng of Cyanine 5 labeled cRNA (carriers or controls) were mixed, fragmented, and hybridized at 65 °C for 17 hours to an Agilent 44 K Whole Human genome Oligo Microarray containing 45,015 features representing 41,000 unique probes. After washing, microarrays were scanned using an Agilent DNA microarray scanner. Feature extraction software (Agilent Technologies, Palo Alto, CA) was used to assess fluorescent hybridization signals and to normalize signals using linear regression and a Lowess curve-fit technique. Reproducibility and reliability of each single microarray was assessed using Quality Control report data (Feature extraction, Agilent Technologies). Self-self and dye swap hybridizations were performed to check data quality and evaluate the importance of dye bias. For self-self hybridizations, aliquots of the same RNA sample were separately labeled with CY3 and CY5 fluorescent dyes and cohybridized to the same microarray.

### Data analysis

Gene expression analysis was carried out using GeneSpring GX software (Agilent Technologies, Palo Alto, CA). Expression ratios were calculated (CY5 processed signal was divided by CY3 processed signal), and normalized per chip to the 50th percentile and finally normalized per gene to medians. We worked on a pre-screened list of 16,997 genes obtained after filtering the data for outliers, negative and positive controls, and on the quality flag CY5 and CY3 signals being “well above background”. To pass this last flag, CY5 and CY3 net signals needed to be positive and significant, with g(r)BGSubSignal greater than 2.6 g(r) BG_SD.

To determine if there were genes differentially expressed between mutation carriers and controls, we performed two Welch’s t-tests (P < 0.01) on this pre-screened list of genes: one without correction and one with Benjamini and Hochberg’s correction. Average linkage hierarchical clustering analysis was applied using Euclidean distance, and differentially expressed genes were annotated using the information from the Gene Ontology Consortium. Panther, Ingenuity Pathways Analysis (Ingenuity Systems®, www.ingenuity.com) and FatiGO software were used to assess whether specific biological processes or molecular functions were differentially expressed, through the over-representation of groups of genes with functional links, rather than individual genes. Global molecular networks and comparison of canonical pathways were generated using Ingenuity Pathways Analysis.

### Allele-specific transcript expression

Single-nucleotide primer extension was performed as described in the Supplementary Methods with the ABI Prism SnaPshot multiplex Kit (Applied Biosystems, Evry, France).

## Results

### Sample characteristics

Sample characteristics are listed in Table 1. We selected a group of fifteen *BRCA1* mutation carriers belonging to 11 distinct high-risk breast and ovarian cancer families and for whom 10 different *BRCA1* mutations were identified by direct sequencing. At the time of blood sample collection, all mutation carriers were healthy and not undergoing treatment, although some of them had had breast or ovarian cancer 3 to 20 years previously. All mutations were deleterious nonsense codons or frameshifts, and were scattered throughout the gene. A comparison group of fifteen healthy relatives without familial *BRCA1* or *BRCA2* mutation was collected. The absence of mutation was verified by direct sequencing for the mutation known to concern each family. Gender distribution was similar between carriers and controls (3 male and 12 female carriers; 4 male and 11 female controls). Age distribution was slightly lower among controls: 57 years for mutation carriers (range 26–76), versus 42 years for controls (range 22–67).

### Distribution of signal intensity and abundance of transcripts

**Figure 1:**
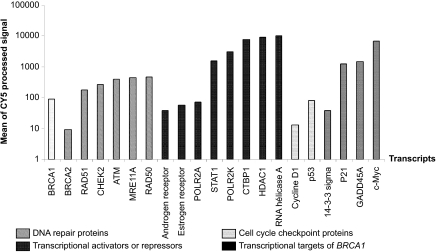
Mean of CY5 processed signal over 30 experiments for selected *BRCA1*-interacting proteins and transcriptional targets.

Signal intensity in lymphocytes was low. Although the dynamic range for the red and green channels was wide (from 30 to 60,000 for net signals), the median intensities were around 80 for both channels. As presented in Figure 1, the average *BRCA1* signal, and therefore expression, was very low. The second major susceptibility gene involved in breast cancer risk, *BRCA2*, was not significantly expressed in PBMCs. Among transcripts coding for BRCA1-interacting proteins, transcriptional regulation proteins were more highly represented than those involved in DNA damage repair or cell cycle checkpoints. Proteins related to estrogen signaling (androgen and estrogen receptors) were not significantly expressed. Most of the known transcriptional targets of *BRCA1* were well represented.

### Unsupervised analysis

**Figure 2:**
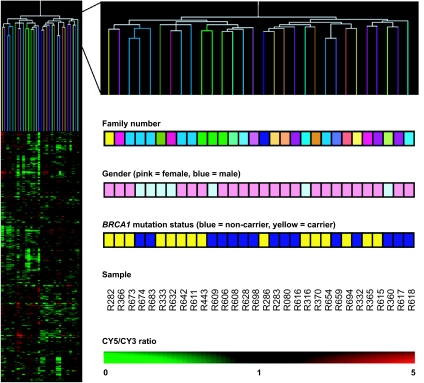
Hierarchical clustering performed on all samples «15 *BRCA1* mutation carriers versus 15 non-carriers» in both the experiment and the gene dimensions using a pre-screened list of 16,997 genes. Branches are color coded according to the family number of each sample.

The mutation carrier and non-carrier samples were cohybridized with an internal reference to Agilent 44 K Whole Human genome Oligo Microarrays. Data were normalized using Feature Extraction software (Agilent Technologies, Palo Alto, CA) and analyzed with Genespring GX software (Agilent Technologies, Palo Alto, CA), resulting in a pre-screened set of 16,997 genes. An unsupervised method was used to reveal distinct clusters according to different parameters, such as *BRCA1* mutation status, gender, age or diagnosis. Average linkage clustering analysis using Euclidean distance was performed in both gene and experiment dimensions. This analysis did not show any clear subgroup of samples with similar expression patterns that associated with *BRCA1* mutation status (Fig. 2). The two main clusters observed in this dendogram were not associated with any of the parameters described above (family number, gender, age, diagnosis, *BRCA1* mutation status). Some samples from the same family grouped together (three samples from family 2001: R673–R674–R683 and two samples from family 1541: R443–R609) regardless of gender or *BRCA1* mutation status, although other samples from the same family were distant in the clustering.

### Supervised analysis

Supervised analysis was performed to identify genes differentially expressed between *BRCA1* mutation carriers and controls, using a t-test based on the *BRCA1* mutation status of each sample on the previous set of 16,997 genes, with a p-value fixed at <0.01. This analysis revealed 133 genes differentially expressed between *BRCA1* mutation carriers and controls.

### Hierarchical clustering

**Figure 3:**
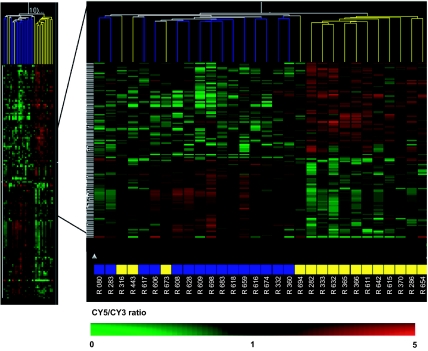
«15 *BRCA1* mutation carriers versus 15 non-carriers» were subjected to hierarchical clustering in both the experiment and the gene dimensions using the 133 differentially expressed genes. Branches are color coded according to the *BRCA1* mutation status of each sample. Blue, non-carriers; Yellow, *BRCA1* mutation carriers.

Hierarchical clustering in both gene and experiment dimensions using these 133 genes (Fig. 3) showed two main clusters with a positive predictive value of 100% and a negative predictive value of 80%. The dendogram branches show eleven of the 15 *BRCA1* mutation carriers grouped together in a first cluster, while the second cluster contains three subgroups in which four *BRCA1* mutation carriers are misclassified with non-carriers. These four samples were not distinguishable from other *BRCA1* mutation carriers by their gender, age, diagnosis, *BRCA1* mutation type or by the functional domain affected by the mutation. None of their characteristics allowed us to exclude them from further analysis.

### Gene list annotation

Among the 133 differentially expressed genes, 105 corresponded to known genes with a unique identifier, and 81 could be classified in a Panther database (listed by function in Table 2). Transcription and translation functions were fairly well represented, with a subunit of RNA polymerase I (POLR1D), a putative RNA helicase (DDX55), and zinc finger transcription factors (ZZEF1, ZFYVE28, PRDM1) tending to be over-expressed in the *BRCA1* mutation carrier group. Immune-response genes were also well represented, with nine genes, including an antigen of the major histocompatibility complex (*HLA-E*), an antibacterial response protein (C1QBP), and a tumor suppressor gene involved in B-cell differentiation (KLF6) differentially expressed. Biological processes linked to other BRCA1 functions such as cell cycle control and DNA repair were less represented. Three genes involved in oncogenesis, including the oncogenes *VAV3* and *YES1*, tended to be up-regulated in the *BRCA1* mutation carriers group.

Using Ingenuity Pathways Analysis software, 67 genes could be used to generate global molecular networks, which identified 13 independent networks mainly involving genes linked to cancer disease. No overlap was observed between these 13 networks with the BRCA1 global molecular network, since no genes were common to our set of 133 genes and the global molecular network of 35 genes connected to BRCA1 and selected from the Ingenuity’s Knowledge database. A search of all molecules upstream and downstream of BRCA1, for all types of relationships, yielded 314 genes linked to BRCA1. Comparison of this list to the 133 differentially expressed genes identified two in common (*DDB2* and *CCL5*), both coding for proteins whose expression was previously shown to be induced by BRCA1.^24^,^25^

The transcript DDB2 codes for Damaged DNA Binding Protein and was shown to be transcription-ally up-regulated by wild type BRCA1 in a p53-dependent manner upon DNA damage.^25^ In our dataset, it tended to be slightly over-expressed in *BRCA1* mutation carriers, even though BRCA1 should be less functional in this group. The other transcript, CCL5, codes for an interferon-inducible gene involved in apoptotic cell death, and has been found to be up-regulated by BRCA1 in breast cancer cell lines.^24^ Like *DDB2*, this gene tended to be slightly overexpressed in *BRCA1* mutation carriers in our dataset.

**Figure 4:**
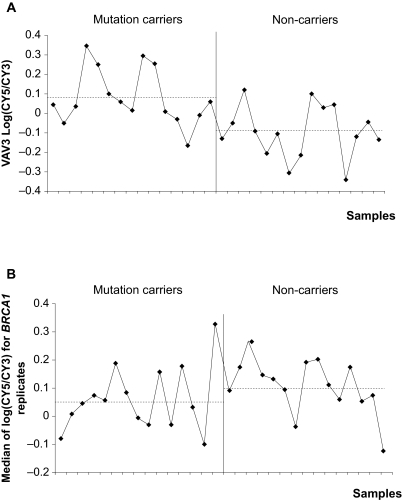
Distribution of log ratios between 15 *BRCA1* mutation carriers and 15 non-carriers for VAV3 and *BRCA1* transcripts. Dashed lines represent mean log ratios in each group (mutation carriers and non-carriers) (**A**) Distribution of log ratios for VAV3 transcript, an oncogene found to be differentially expressed between mutation carriers and non-carriers after a t-test with a p-value < 0.01 performed on 16,997 genes. (**B**) Distribution of median log ratios for the ten *BRCA1* replicates.

None of the other BRCA1-interacting proteins and transcriptional targets cited previously and presented in Figure 1 were present among the 133 differentially expressed genes (Table 2). Fold-changes in gene expression for differentially expressed genes had little amplitude, and the standard deviation within the same group was large. As an example, in Figure 4A, although the VAV3 transcript tended to be slightly more represented in *BRCA1* mutation carriers, the log ratio variation between the two groups was significant but weak.

### Supervised analysis with correction of false discovery rate

In order to limit the number of significant genes due to random chance among the 16,997 tested genes, we performed a t-test with Benjamini and Hochberg multiple testing correction with a p-value less than 0.01. This analysis did not show any genes differentially expressed between *BRCA1* mutation carriers and controls.

### Supervised analysis of BRCA1-interacting proteins and targets

Expression profiles of 52 BRCA1-interacting proteins and transcriptional targets were compared to mutation status. None of these genes appeared significant in a t-test with a p-value less than 0.05. Two genes, *STAT1* and *TERT,* had p-values less than 0.10. *BRCA1* gene expression levels are shown in Figure 4B; no significant changes in gene expression were observed (p = 0.16).

## Discussion

We compared gene expression profiles of untreated PBCMs from 15 *BRCA1* mutation carriers and 15 non-carriers. Of 16,997 genes tested, statistical analysis revealed 133 to be differentially expressed at p ≤ 0.01. This number was smaller than the approximately 170 genes expected by random chance. Hierarchical clustering performed on the 133 differentially expressed genes revealed four *BRCA1* mutation carrier samples misclassified in the non-carrier group. Among this list of differentially expressed transcripts, 60% could be annotated through Panther and FatiGO databases: these were mainly involved in cellular metabolic processes and to a lesser extent in immune response and transcription. There was a weak variation in their log-ratio expression between *BRCA1* mutation carriers and non-carriers. Although supervised analysis revealed a tendency for these genes to be differentially expressed in *BRCA1* mutation carriers, these genes could not be used to define a robust and reliable signature for *BRCA1* heterozygosity in PBMCs. The variation in expression was too weak between carriers and controls, and they did not allow us to discriminate all *BRCA1* mutation carrier samples from *BRCA1* or *BRCA2* non mutation carrier samples.

Considering the very large number of genes tested (nearly 17,000) and the small number of samples (30), it is likely that random chance will yield some genes which are not really significant even though they appear to discriminate between the two populations. Using a more stringent test to control this false discovery rate, we did not find any genes passing this statistical restriction filter. Moreover, *BRCA1* itself had low signal intensity in PBMCs and, like its partners and transcriptional targets, did not show any significant changes in gene expression correlated to its mutation status.

**Supplementary Figure 1:**
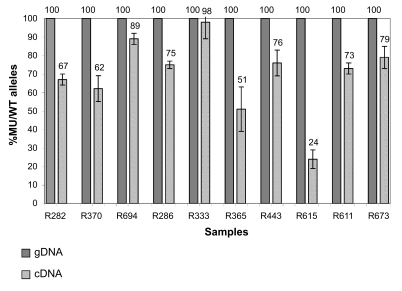
Relative abundance of mutant “MU” versus wild type “WT” *BRCA1* alleles expressed in peripheral blood lymphocytes. Quantitative analysis of allelic ratios in mRNA and genomic DNA (gDNA) was performed using the SnaPshot technique (Applied Biosystems, Foster City, CA). Alleles were discriminated with heterozygous single nucleotide polymorphisms present in the coding sequence of *BRCA1*. Normalization was performed by dividing the observed values by those obtained for the corresponding genomic DNA.

This lack of difference in gene expression patterns between *BRCA1* mutation carriers and controls could be due in part to an mRNA surveillance pathway, Nonsense Mediated Decay (NMD), which eliminates mRNAs harboring truncating mutations, thus limiting the production of truncated proteins with downstream deleterious effects. The majority of the mutant *BRCA1* transcripts were tested for NMD (Supplementary Fig. 1) in PBMCs and most showed significantly reduced levels of the mutant allele compared to the wild-type allele. This reduction of *BRCA1* mutant transcript may limit any deleterious effects of mutant BRCA1 protein on its transcriptional targets or partners, resulting in a recessive effect at the cellular level. This elimination of the mutant transcript, however, did not result overall in detectably lower levels of expression of BRCA1 itself; it seems that inter-individual variation was too great for direct detection of mutation carriers. This inter-individual variation could be due to confounding factors, such as time of blood sampling, menstrual cycle phase, stress, dietary patterns and/or intake of medications.

Another source of error to consider is 3′-end bias. Reverse-transcription using oligo-dT primers biases this study in favor of detecting the 3′ end of transcripts in the hybridization step, and is not suitable for detecting variants alternatively spliced far upstream of the 3′ end. To address this issue, other strategies could be employed, notably random priming of the RT-PCR reaction,^26^ and the use of exon-specific arrays, in which probes designed to interrogate variant transcripts are included in the array.

Comparing our results with microarray data from other groups obtained after irradiation confirms that *BRCA1* is a response gene, and a stimulus such as DNA damage is necessary to reveal the phenotype. This haploinsufficiency is not detectable in the absence of exceptional stress. Cancer risk associated with *BRCA1* mutation can thus be explained by two models. First, random loss of the wild-type allele in sensitive tissues such as breast and ovary results in a small population of BRCA1-null cells, which are now highly susceptible to oncogenesis. This model is borne out by the observation that loss of the wild-type allele is indeed a very common and early step in breast oncogenesis in mutation carriers. A second, non-exclusive model proposes that a single allele of BRCA1 is sufficient for normal cellular metabolism, but is insufficient to adequately respond to genotoxic stress. Irradiation thus reveals a phenotype not otherwise detectable. The sub-normal response to DNA damage may result in the fixation of mutations and the early steps of oncogenesis.

Previous studies demonstrate that gene expression profiles can be a powerful tool to predict *BRCA1* mutation status in malignant tissue or in irradiated healthy tissue.^8^,^10^,^12^,^14^,^15^ However, the different studies rarely retain the same differentiating genes and a large number of false positives are to be expected due to the small population sizes.^27^ By examining gene expression profiles of *BRCA1* mutation carriers and non-carriers in untreated PBMCs, it seems difficult to accurately distinguish carriers from non-carriers. This lack of a sufficiently robust *BRCA1* mutation carrier signature in untreated samples unfortunately inhibits the development of a pre-screening tool based on samples that are drawn at some time and distance from the analyzing laboratory or which for other reasons cannot undergo treatment appropriate to reveal the heterozygous phenotype.

## Supplementary Data

### Allele-specific transcript expression

Genomic DNA was extracted from peripheral blood using standard methods. Samples were genotyped for two common single nucleotide polymorphisms (SNPs): c.2612C>T (SNP ID rs 799917, located in exon 11 of *BRCA1*) and c.4837A>G (SNP ID rs 1799966, located in exon 16 of *BRCA1*). We selected samples that were heterozygous for one of these polymorphisms (ten out of fifteen). Complementary cDNA was synthesized from 1–2.5 μg total RNA using RT2 PCR Array first strand kit (SuperArray Bioscience Corporation, Frederick, MD). Before reverse transcription, a genomic DNA elimination step was performed according to the manufacturer’s instructions. Amplification primers (shown in Supplementary Table 1) were designed using Primer Express software (Applied biosystems, Evry, France) so that they would surround these SNPs and were provided by MWG Biotech (Roissy, France). Amplification was performed using reagents purchased from Applied Biosystems (Evry, France) in a Primus HT thermocycler (MWG Biotech, Roissy, France). PCRs were carried out in a final volume of 10 μl containing 50 ng of gDNA or 75 ng of cDNA, 0.4 μM reverse and forward primers, (each deoxynucleotide triphosphate at 400 μM), 1.5 mM MgCl2 contained in 10X PCR Buffer, 0.6 U AmpliTaq DNA polymerase (Applied biosystems, Evry, France). Thermocycling conditions were: 94 °C for 5 min followed by 30 cycles of 94 °C for 20 sec, 54 °C for 20 sec and 72 °C for 20 sec with a final extension step of 72 °C for 7 min. PCR products were purified in a one-step reaction by the addition of 1 μl of ExoSap reagent (Applied biosystems, Evry, France) to 5 μl of PCR products in a final volume of 7 μl. The mixture was incubated at 37 °C for 30 min, followed by enzyme deactivation at 80 °C for 30 min. Purified PCR products were then analyzed using a primer extension method (SNaPshot). Extension primers (shown in Supplementary Table 1) were designed to anneal to the amplified DNA template immediately adjacent to the heterozygous single nucleotide polymorphim site. Single nucleotide primer extension was performed in a final volume of 9 μl with 2 μl of purified PCR products, 3 μl of SNaPshot reaction mix (Applied biosystems, Evry, France) and 0.17 μM of a specific primer related to the heterozygous SNP analyzed. After purification through a sephadex column, the extended primers labelled with different fluorescent dyes were run on an ABI 3100 capillary electrophoresis instrument and analyzed with GeneMapper software (Applied biosystems, Evry, France). Peak area ratios were calculated to measure the relative amount of the two alleles for cDNA and genomic DNA. Normalization was performed by dividing the peak area ratios obtained for cDNA by those obtained for the corresponding genomic DNA which was defined as 1. Three independent experiments (PCR and SNaPshot reactions) were performed for each sample.

## Figures and Tables

**Table 1:** Sample characteristics for *BRCA1* mutation carriers and non-carriers.

Sample	Family number	Sex	Age	Diagnosis (Age)	Familial *BRCA1* mutation	Carrier Yes/No	Mutation type	Exon
R282	0017–01	F	74	Breast cancer (54)	c. 3841_3843delCA	Yes	Frameshift	11
R370	0278–30	F	48	No cancer	C.3607C>T	Yes	Stop	11
R694	0401–36	F	26	No cancer	c. 68_69delAG	Yes	Frameshift	2
R632	0815–14	M	76	No cancer	c. 4810C>T	Yes	Stop	16
R286	0922–01	F	59	Breast cancer (51)	C.178C>T	Yes	Stop	5
R333	0929–09	M	56	No cancer	c. 4248_4249 del TG	Yes	Frameshift	13
R316	1197–08	M	44	No cancer	c. 3839_3843delins4	Yes	Frameshift	11
R365	1447–01	F	73	Breast cancer (70)	c. 4282ins41	Yes	Frameshift	13
R366	1447–06	F	51	Breast cancer (37)	c. 4282ins41	Yes	Frameshift	13
R443	1541–01	F	59	Breast cancer (50)	c. 4163dupA	Yes	Frameshift	12
R615	1971–03	F	76	No cancer	c.4065_4068delTCAA	Yes	Frameshift	11
R642	2001–01	F	67	No cancer	c. 3839_3843delins4	Yes	Frameshift	119
R611	2001–08	F	68	Breast cancer (59)	c. 3839_3843delins4	Yes	Frameshift	119
R673	2001–35	F	44	No cancer	c. 3839_3843delins4	Yes	Frameshift	119
R654	2001–59	F	33	No cancer	c. 3839_3843delins4	Yes	Frameshift	119
R080	0080–38	F	40	No cancer	None*	No		
R360	0271–52	M	67	No cancer	None*	No		
R608	0554–46	F	45	No cancer	None*	No		
R606	0719–20	F	41	No cancer	None*	No		
R698	0822–19	F	58	No cancer	c. 4810C>T	No		
R618	0998–38	F	32	No cancer	c. 3839_3843delins4	No		
R283	1119–01	F	38	No cancer	None*	No		
R332	1212–43	F	36	No cancer	c. 178C>T	No		
R659	1317–08	F	22	No cancer	None*	No		
R628	1393–08	F	38	No cancer	c. 1504_1508del5	No		
R609	1541–10	M	60	No cancer	c. 4163dupA	No		
R617	1971–01	F	45	No cancer	c. 4065_4068delTCAA	No		
R616	1971–16	F	37	No cancer	c. 4065_4068delTCAA	No		
R674	2001–36	M	36	No cancer	c. 3839_3843delins4	No		
R683	2001–80	M	34	No cancer	c. 3839_3843delins4	No		

Non-carriers are healthy relatives tested negative by direct sequencing for a known *BRCA1* or *BRCA2* mutation present in their family. Only familial *BRCA1* mutations are described in this table.

* None: When non-carriers belong to a *BRCA2* family, the *BRCA2* familial mutation is not described.

**Table 2:** Gene list for 81 annotated genes selected from the 133 transcripts found to be differentially expressed between *BRCA1* mutation carriers and non carriers after t-test with a p value < 0.01.

Accession number	Gene name	Gene symbol	T-test P-value	Fold change
(1) Transcription and translation				
(1.1) Transcription				
A_23_P409541	Polymerase (RNA) I polypeptide D, 16 kDa	POLR1D	0,00886	−1,18
A_32_P104746	Zinc finger, FYVE domain containing 28	ZFYVE28	0,00136	1,35
A_24_P282108	Zinc finger, ZZ-type with EF-hand domain 1	ZZEF1	0,00355	1,16
A_32_P169550	PR domain containing 1, with ZNF domain	PRDM1	0,00742	1,81
A_32_P57717	Leucine rich repeat (in FLU) interacting protein 1	LRRFIP1	0,00969	1,40
(1.2) RNA interaction and protein synthesis				
A_23_P257609	Ribosomal protein L29	RPL29	0,0033	−1,27
A_24_P538567	Ribosomal protein L15	RPL15	0,00583	−1,34
A_32_P76399	Eukaryotic translation initiation factor 3, subunit 6 interacting protein	EIF3S6IP	0,00829	−1,13
A_23_P47839	DEAD (Asp-Glu-Ala-Asp) box polypeptide 55	DDX55	0,00908	−1,13
A_24_P916251	Ribosomal protein L28	RPL28	0,00947	−1,29
A_23_P202071	CUG triplet repeat, RNA binding protein 2	CUGBP2	0,00334	1,35
A_24_P205008	tRNA splicing endonuclease 54 homolog (*S. cerevisiae*)	TSEN54	0,00534	1,42
A_23_P86943	Signal recognition particle receptor (‘docking protein’)	SRPR	0,00678	1,17
(2) Immunity and defense				
A_23_P74290	Guanylate binding protein 5	GBP5	0,0015	−1,56
A_23_P171255	Immunoglobulin (CD79A) binding protein 1	IGBP1	0,00373	−1,17
A_23_P370434	Complement component 1, q subcomponent binding protein	C1QBP	0,00937	−1,15
A_24_P315986	Major histocompatibility complex, class I, E	HLA-E	0,00961	−1,16
A_23_P201551	Vav 3 oncogene	VAV3	0,0017	1,19
A_23_P104193	Integrin, beta 1 (fibronectin receptor, beta polypeptide, antigen CD29 includes MDF2, MSK12)	ITGB1	0,00469	1,27
A_23_P152838	Chemokine (C-C motif) ligand 5	CCL5	0,00579	1,52
A_24_P252739	Kruppel-like factor 6	KLF6	0,00727	1,25
A_24_P48403	V-yes-1 Yamaguchi sarcoma viral oncogene homolog 1	YES1	0,00991	1,37
(3) Protein folding and degradation				
A_23_P18604	Leucine aminopeptidase 3	LAP3	0,00624	−1,30
A_23_P79911	Proteasome (prosome, macropain) inhibitor subunit 1 (PI31)	PSMF1	0,0079	−1,11
A_24_P127021	Suppression of tumorigenicity 13(colon carcinoma) (Hsp70 interacting protein)	ST13	0,00971	−1,22
A_24_P174613	F-box and WD-40 domain protein 7 (archipelago homolog, Drosophila)	FBXW7	0,00248	1,22
A_24_P139191	Itchy homolog E3 ubiquitin protein ligase (mouse)	ITCH	0,00584	1,29
A_23_P16409	Calpain 12	CAPN12	0,00767	1,42
A_23_P73992	Ubiquitin specific peptidase 24	USP24	0,00924	1,20
(4) Intracellular protein traffic				
A_23_P20045	peroxisome biogenesis factor 1	PEX1	0,00267	1,16
A_24_P248240	Synaptotagmin XI	SYT11	0,00408	1,35
A_23_P72627	Hepatocyte growth factor-regulated tyrosine kinase substrate	HGS	0,00507	1,19
A_23_P165952	ARP5 actin-related protein 5 homolog (yeast)	ACTR5	0,00751	1,15
A_32_P122268	Synaptotagmin XV	SYT15	0,00983	1,43
(5) Signaling and signal transduction				
A_32_P207360	Adenylate kinase 2	AK2	0,00532	−1,17
A_23_P125164	Fragile histidine triad gene	FHIT	0,00675	−1,33
A_23_P14915	Casein kinase 2, alpha prime polypeptide	CSNK2A2	0,00988	−1,19
A_23_P135184	Ral guanine nucleotide dissociation stimulator	RALGDS	0,0039	1,36
A_23_P259611	Thioredoxin domain containing 3 (spermatozoa)	TXNDC3	0,00632	1,37
(6) Cell cycle				
A_23_P43157	V-myb myeloblastosis viral oncogene homolog (avian)-like 1	MYBL1	0,00354	1,54
A_23_P163178	Calmodulin 1 (phosphorylase kinase, delta)	CALM1	0,00642	1,15
A_24_P50458	Telomeric repeat binding factor (NIMA-interacting) 1	TERF1	0,00652	1,18
A_24_P361896	Protein phosphatase 1, catalytic subunit, beta isoform	PPP1CB	0,00839	1,34
(7) DNA repair and modification				
A_23_P259641	Enhancer of zeste homolog 2 (*Drosophila*)	EZH2	0,00212	1,21
A_23_P52610	Damage-specific DNA binding protein 2, 48 kDa	DDB2	0,00307	1,20
A_23_P416468	PIF1 5′-to-3′ DNA helicase homolog (*S. cerevisiae*)	PIF1	0,00992	1,41
(8) Other				
A_23_P94736	ST6 (alpha-N-acetyl-neuraminyl-2, 3-beta-galactosyl-1,3)-N-acetylgalactosaminide alpha-2,6-sialyltransferase 4	ST6GALNAC4	0,000288	−1,23
A_23_P80643	SET domain and mariner transposase fusion gene	SET MAR	0,00124	−1,20
A_23_P22765	NADH dehydrogenase (ubiquinone) 1 beta subcomplex, 11, 17.3 kDa	NDUFB11	0,00231	−1,25
A_24_P152635	Thioredoxin domain containing 14	TMX2	0,00378	−1,14
A_23_P204052	Poly(rC) binding protein 2	PCBP2	0,00686	−1,18
A_23_P155103	Adenylosuccinate lyase	ADSL	0,00701	−1,20
A_23_P29046	Carbonyl reductase 1	CBR1	0,00769	−1,29
A_23_P35456	SH3 and PX domains 2A	SH3MD1	0,00998	−1,34
A_23_P151415	Katanin p60 subunit A-like 1	KATNAL1	0,000562	1,36
A_24_P411899	Ring finger protein 19	RNF19	0,00182	1,27
A_23_P19102	UDP-N-acetyl-alpha-D-galactosamine: polypeptide N-acetylgalactosaminyltransferase 10 (GalNAc-T10)	GALNT10	0,00233	1,29
A_32_P216004	Potassium channel tetramerisation domain containing 9	KCTD9	0,00342	1,27
A_23_P109201	Chromosome 20 open reading frame 3	C20orf3	0,00662	1,28
A_23_P80739	Phospholipase C, delta 1	PLCD1	0,00723	1,20
A_24_P106057	Phosphate cytidylyltransferase 1, choline, alpha	PCYT1A	0,00937	1,13
(9) Unclassified				
A_23_P209337	Family with sequence similarity 119, member A	FAM119A	0,0017	−1,39
A_23_P121956	tRNA-histidine guanylyltransferase 1-like (*S. cerevisiae*)	ICF45	0,0024	−1,21
A_23_P105794	Epithelial stromal interaction 1 (breast)	EPSTI1	0,00407	−1,38
A_23_P401098	Chromosome 18 open reading frame 17	C18orf17	0,00491	−1,23
A_23_P32444	Matrix-remodelling associated 8	MXRA8	0,00533	−1,89
A_23_P78677	Chromosome 19 open reading frame 53	C19orf53	0,00609	−1,21
A_23_P370625	Selenoprotein N, 1	SEPN1	0,00673	−1,26
A_23_P6762	Jagunal homolog 1 (*Drosophila*)	JAGN1	0,00678	−1,13
A_23_P258837	Transmembrane protein 142A	ORAI1	0,0075	−1,17
A_24_P340866	Coiled-coil domain containing 32	CCDC32	0,00912	−1,21
A_23_P112004	Leucine rich repeat containing 6	LRRC6	0,00064	1,51
A_24_P240166	Pleckstrin homology-like domain, family B, member 2	PHLDB2	0,00206	1,59
A_32_P18723	Transmembrane protein 64	TMEM64	0,00243	1,22
A_23_P106844	Metallothionein 2A	MT2A	0,00332	1,17
A_23_P206724	Metallothionein 1E (functional)	MT1E	0,00487	1,35
A_23_P500861	Spectrin repeat containing, nuclear envelope 1	SYNE1	0,00533	1,25
A_23_P79628	Proteasome (prosome, macropain) activator subunit 4	PSME4	0,00747	1,19
A_24_P944154	Multiple C2 domains, transmembrane 2	MCTP2	0,00837	1,76
A_23_P40548	Yippee-like 1 (Drosophila)	YPEL1	0,00876	1,26
A_23_P78383	Chromosome 18 open reading frame 8	C18orf8	0,0089	1,16

Genes are classified into 9 categories according to their function. Fold change indicates the relative change between the mean of *BRCA1* mutation carriers expression ratios and the mean of non-carriers expression ratios. Negatives fold changes correspond to genes down regulated in *BRCA1* mutation carriers group and positives fold changes correspond to genes up regulated in *BRCA1* mutation carriers group.

**Table S1:** Primers used for PCR and single nucleotide primer extension (SNaPshot).

Primer name	Sense	Reaction	Sequence	Template	Polymorphism
BRCA1_ex115F	Forward	PCR	5′-TCAAGGTTTCAAAGCGCCA-3′	gDNA, cDNA	Rs 799917
BRCA1_ex115R	Reverse	PCR	5′-TTACGGCTAATTGTGCTCACTGTACT-3′	gDNA, cDNA	Rs 799917
BRCA1_ex16F	Forward	PCR	5′-AATTCTTAACAGAGACCAGAAC-3′	gDNA	Rs 1799966
BRCA1_ex16R	Reverse	PCR	5′-AAAACTCTTTCCAGAATGTTGT-3′	gDNA	Rs 1799966
BRCA1_ex15–16F	Forward	PCR	5′-GCAAGATCTAGAGGGAACCCC-3′	cDNA	Rs 1799966
BRCA1_ex16′R	Reverse	PCR	5′-GGCTTCTCCCTGCTCACACT-3′	cDNA	Rs 1799966
SNP_BRCA1_Rs 17F	Forward	SNaPshot	5′-AAAGCGCCAGTCATTTGCTC-3′	gDNA, cDNA	Rs 799917
SNP_BRCA1_Rs 66R	Reverse	SNaPshot	5′-CAGTAGTATGAGCAGCAGCTGGAC-3′	gDNA, cDNA	Rs 1799966
